# Hypomethylation of Rnase6 Promoter Enhances Proliferation and Migration of Murine Aortic Vascular Smooth Muscle Cells and Aggravates Atherosclerosis in Mice

**DOI:** 10.3389/fbioe.2021.695461

**Published:** 2021-07-28

**Authors:** YongPeng Fang, JinShuang Li, XuDong Niu, NingShun Ma, Jia Zhao

**Affiliations:** ^1^ Department of Cardiovascular Medicine, General Hospital of Ningxia Medical University, Yinchuan, China; ^2^ Department of Cardiology, Suqian Hospital Affiliated to Xuzhou Medical University, Suqian, China; ^3^ Department of Internal Medicine, Yinchuan Women and Children Healthcare Hospital, Yinchuan, China; ^4^ Department of Laboratory Medicine, Xi’an Central Hospital, Xi’an, China

**Keywords:** atherosclerosis, ribonuclease 6, hypomethylation, murine aortic vascular smooth muscle cells, proliferation, migration

## Abstract

**Background:** Accumulating evidence has implicated DNA methylation in the progression of atherosclerosis (AS). Rnase6 has been reported to be upregulated in AS development, but the specific regulatory mechanism remains unclear.

**Material/Methods:** Peripheral blood and sclerotic plaque tissues from 25 AS patients were collected to detect Rnase6 expression. Methylation-specific polymerase chain reaction (MSP) was used to detected Rnase6 methylation levels in the peripheral blood of AS patients. Rnase6 expression was knocked down or DNA methyltransferase 1 (DNMT1) was overexpressed in OX-LDL-treated mouse aortic smooth muscle cells (MOVAS), and cell proliferation, migration, ROS content, and inflammatory factor secretion levels were detected. 740 Y-P, a PI3K specific agonist, was introduced to verify the effect of Rnase6 promoter hypomethylation on the PI3K/Akt signaling pathway. We knocked down Rnase6 expression in ApoE^−/−^ mice fed with a high-fat diet to examine Rnase6 promoter methylation levels. Plaque areas and inflammatory factor secretion were examined in AS mice overexpressing DNMT1.

**Results:** Rnase6 expression was upregulated in the peripheral blood and plaque tissues of AS patients, accompanied by decreased methylation levels of the Rnase6 promoter. Interfering with Rnase6 expression or overexpressing DNMT1 in OX-LDL stimulated MOVAS inhibited cell proliferation and migration, decreased ROS content and inflammatory factor secretion, and inhibited PI3K pathway protein expression. Rnase6 expression was decreased in the peripheral blood and plaque tissues of si-Rnase6-injected mice, and Rnase6 promoter methylation was increased. Mice overexpressing DNMT1 showed less plaque areas in the aortic root and lower secretion levels of inflammatory factors.

**Conclusion:** Hypomethylation of the promoter of Rnase6 enhanced the proliferation and migration of OX-LDL treated MOVAS, upregulated ROS content and inflammatory factor secretion levels in the cells, and activated the PI3K/Akt signaling pathway.

## Introduction

Atherosclerosis (AS) is a chronic inflammatory disease caused by multiple factors. It is characterized by vascular endothelial dysfunction, subintimal lipid deposition, abnormal migration and proliferation of smooth muscle cells, and foam cell formation. It can involve the arterial blood vessels of important organs of the whole body, in which a variety of vascular cells such as endothelial cells, smooth muscle cells, macrophages and lymphocytes are involved in the occurrence of AS (Lacey et al., 2019; Gao, et al., 2019; Sun, et al., 2019). Studies have shown that dysfunction of endothelial cells (ECs) leading to the deposition of cholesterol and other lipids in the vessel wall is the initial event leading to the occurrence of AS (Viafara-Garcia, et al., 2018; Deng, et al., 2018). Damage to ECs also leads to the invasion of proinflammatory factors into the intima and abnormal proliferation of vascular smooth muscle cells (VSMCs) (Baumer, et al., 2017; Li, et al., 2017; Yu, et al., 2018). During the progression of AS, VSMCs undergo dedifferentiation, proliferation, migration under stress conditions, and promote fibrous cap formation. VSMCs can transform into macrophage like cells that are proinflammatory and dysfunctional and “synthetic” smooth muscle cells that can produce extracellular matrix, increasing the risk of myocardial infarction.

Increasing evidence indicates that epigenetics, such as DNA methylation, acetylation, glycosylation and phosphorylation, play an important role in the occurrence and development of many diseases and regulate gene expression (Abdel-Hafiz and Horwitz, 2015; Wu, et al., 2020). Among them, DNA methylation refers to the methyl group in S-adenosylmethionine is transferred to cytosine by DNA methyltransferase (DNMT) to form 5-methylcytosine, which is methylated (Guarrera, et al., 2019; Jamebozorgi, et al., 2018). DNA methylation in humans and mammals is mainly associated with CpG dinucleotides, and hypermethylation of CpG islands in the promoter region can inhibit gene expression. In recent years, a large number of studies have shown that DNA methylation and other factors are involved in the regulation mechanism of atherosclerosis. Wei et al. found that SMAD7 expression was decreased and its promoter region was significantly methylated in atherosclerotic plaques compared with normal arterial walls. And increased DNA methylation levels of the SMAD7 promoter at CpG unit 5.8.15.16 were found in the peripheral blood of patients with atherosclerosis compared with normal controls, suggesting that methylated SMAD7 may be a novel predictive marker for atherosclerosis (Wei, et al., 2018). In addition, Xia et al. identified 174 upregulated genes with hypomethylation in the promoter and 86 downregulated genes with hypermethylation in the promoter in as vs. healthy controls, found that signaling lymphocyte activation molecule 7 (SLAMF7) was expressed at high levels in carotid plaques, and the expression of SLAMF7 protein was significantly higher in unstable plaques than in stable plaques (Xia, et al., 2018).

Recently, researchers performed comprehensive bioinformatics analysis on specimens from 16 advanced plaques and 13 early plaques, identified differentially expressed genes, and obtained protein-protein interaction networks. Among them, Ribonuclease 6 (Rnase6) was reported to occur in AS with up-regulated expression (Zhao, et al., 2020). Rnase6 belongs to the secreted protein of the Ribonuclease A (RNaseA) superfamily and has a wide range of physiological functions, including digestion, cytotoxicity, angiogenesis, male reproduction and host defense (Lang, et al., 2019). It has been reported that Rnase6 shows higher expression in uterine tissues with abnormal placental expulsion after delivery, leading to macrophage polarization toward M2 phenotype and aggravating the inflammatory process during placental expulsion (Nelli, et al., 2019). However, the specific regulatory mechanism of Rnase6 in the process of atherosclerosis is still unclear and needs further exploration.

In this study, we used promoter methylation of Rnase6 as an entry point to explore the specific regulatory mechanisms of decreased promoter methylation of Rnase6 on cell proliferation, migration, oxidative stress and inflammation in MOVAS cells treated with OX-LDL, to understand the role of Rnase6 in the development and progression of AS, and to provide a new reference for evaluating the potential therapeutic potential of Rnase6 for AS treatment.

## Materials and Methods

In this study, we examined the expression levels of Rnase6 in the peripheral blood of healthy volunteers and patients with AS, as well as in plaque tissues and surrounding healthy arteries from patients with AS. Next, MOVAS cells were treated with OX-LDL, and the levels of Rnase6 expression, cell proliferation, migration, oxidative stress, inflammatory factor secretion, and PI3K signaling pathway protein expression were examined. We predicted the presence of a CpG island at the mouse Rnase6 gene promoter by online bioinformatics databases, and ChIP experiments confirmed DNMT1 binding to Rnase6. DNMT1 was overexpressed in MOVAS cells treated with OX-LDL, and the levels of Rnase6 expression, cell proliferation, migration, oxidative stress, secretion of inflammatory factors, and PI3K signaling pathway protein expression were examined. An ApoE knockout mouse model with knockdown of Rnase6 expression was constructed, and the Rnase6 expression and methylation levels in mouse peripheral blood were detected. An ApoE knockout mouse model overexpressing DNMT1 was constructed to determine the expression levels of Rnase6 and inflammatory factors in aortic root tissues, and the plaque areas in aortic root were observed by HE staining.

### Samples

Atherosclerotic plaque tissue specimens and adjacent healthy arterial wall specimens were obtained from patients undergoing carotid endarterectomy (*n* = 25, 69.35 ± 4.2 years). And 10 ml of peripheral venous blood of patients was collected for the study the day before surgical treatment. In addition, atherosclerotic plaques and healthy arteries were obtained from patients undergoing carotid endarterectomy. The collected samples were washed in precooled PBS (0.02 mol/L, pH 7.0–7.2) to remove blood and added to 5 ml PBS for thorough grinding. The prepared homogenates were homogenized in 5,000 × g centrifuged for 5 min, and the supernatant was retained and used for subsequent studies. The study protocol was approved by the Ethics Committee of Xi’an Central Hospital, followed the Declaration of Helsinki and other bioethical principles, and obtained written informed consent from each participant.

### Cell Culture

Mouse aortic vascular smooth muscle cells were purchased from the American Type Culture Collection (ATCC, Manassas, VA, United States). Cells were incubated with 10% fetal bovine serum and 100 U/mL penicillin and 100 μg/ml streptomycin (Sigma,St. Louis, MO, United States) in RPMI 1640 medium (Gibco, Rockville, MD) containing 5% CO_2_. Small interfering RNA (si-Rnase6, 30 nM) and negative control of Rnase6 (scramble) were purchased from Santa Cruz Biotechnology (Santa Cruz, CA, United States). The siRNA sequences were as follows: for sense Rnase6 siRNA: 5′-GGA GCU AGC UGU UAG CUA AUU-3ʹ, for antisense Rnase6 siRNA: 5′-UUA GCU AAC AGC UAG CUC CCA-3′; for control siRNA (scramble): 5-UUC UCC GAA CGU GUC ACG UTT-3. The overexpression plasmid of DNMT1 (pcDNA-DNMT1, 2 μg/ml) was purchased from RiboBio Co., Ltd. (Guangzhou, China). All reagents were transfected into cells using Lipofectamine 3,000 transfection reagent (Invitrogen, Carlsbad, CA, United States) according to the manufacturer’s instructions. In addition, OX-LDL treatment of mouse aortic vascular smooth muscle cells for 48 h induced foam cell formation.

### Animals

Eight-week-old C57BL/6 pure line ApoE gene knockout (ApoE^−/−^) mice were purchased from Guangdong Animal Experimental Center. The ratio of male to female was 1:1, and all animals were housed in separate cages in temperature and light cycle-controlled environments with free access to food and water. Sixteen ApoE^−/−^ mice were divided into control group (ApoE^−/−^ mice were given a basal diet), model group (ApoE^−/−^ mice were given a high-fat diet), scramble group (scramble was injected into the tail vein of high fat diet fed ApoE^−/−^ mice, 0.5 mg/kg, twice a week) and si-Rnase6 group (si-Rnase6 was injected into the tail vein of high fat diet fed ApoE^−/−^ mice, 0.5 mg/kg, twice a week), with four mice in each group. Besides, eight ApoE^−/−^ mice were divided into pcDNA3.1 group (pcDNA3.1 was injected into the tail vein of high fat diet fed ApoE^−/−^ mice, 200 μg/ml) and pcDNA-DNMT1 group (pcDNA-DNMT1 was injected into the tail vein of high fat diet fed ApoE^−/−^ mice, 200 μg/ml), with four mice in each group. High-fat diet feeding protocol: mice were fed with a diet containing 1.25% cholesterol, 0.5% cholic acid, and 15% fat for 10 weeks. All animal care and experimental procedures were approved by the Ethics Committee of Xi’an Central Hospital.

### CCK-8 Assay

The murine aortic vascular smooth muscle cells cultured for the indicated periods of time (0–72 h), and cell viability was detected using the Cell Counting Kit-8 (CCK-8) according to the manufacturer’s instructions (Beyotime Institute of Biotechnology, Shanghai, China) and read using a microplate reader (Synergy HT, BioTek), at 450 nm.

### ELISA

The secretion level of Rnase6 in peripheral blood of atherosclerotic patients and mice was determined by ELISA kits (Sigma, St. Louis, MO, United States) according to the manufacturer’s instructions. Similarly, the secretion levels of TNF-α, IL-6 and IL-1β in cell culture supernatants were also detected by ELISA kits.

### ROS Content Detection

MOVAS were plated at 1.5 × 10^5^ density seeded in culture flasks. MOVAS without any treatment were used as the control group. MOVAS were treated with OX-LDL (80 μg/ml) alone for 24 h, or transfected together with si-Rnase6 for 48 h. ROS kit (S0033, Beyotime, Shanghai, China) was used to measure ROS levels according to the manufacturer’s instructions. Dichloro-dihydro-fluorescein diacetate (DCFH-DA; 10 μM) was added to the cells and incubated for 20 min at 37°C. The cells were then digested and suspended. The cell suspensions were centrifuged at 1,000 × g for 10 min and washed twice with phosphate-buffered saline (PBS). The cells were collected after centrifugation for fluorescence detection. Flow cytometry was used to measure fluorescence intensity. The positive area of DCFH-DA was ROS fluorescence intensity.

### Chromatin Immunoprecipitation

EZ-Magna ChIP TMA kit (Millipore, Billerica, MA) were employed for chromatin immunoprecipitation (ChIP) analysis following the manufacturer’s guideline. Briefly, MOVAS were cross-linked with 1% formaldehyde for 10 min, and then 125 mM glycine was added to terminate the crosslinking. Next, cells were lysed in lysis buffer, and chromatin fragments at 200–1,000 bp were obtained by cracking the cells through ultrasound. The supernatant was centrifuged and the fragments were collected in three tubes, which were supplemented with the target protein specific antibody (DNMT1, ab13537, Abcam) or the negative control antibody (IgG, ab10948, Abcam) for incubation at 4°C overnight. The DNA-protein complex was precipitated with Protein Agarose/Sepharose. The precipitated DNA fragments were purified and subjected to RT-qPCR analysis.

### Western Blotting

Protein homogenates from cells were extracted. The specific operation was as follows: the cells were collected and rinsed with PBS for 2 times, and 10 ml culture solution was added after trypsinization to prepare a cell suspension. The cell suspension was moved into a centrifuge tube at 4°C, 2,500 rpm, and the supernatant was discarded after centrifugation for 5 min, repeated twice. Then 200 μl cell lysate was added to the cell suspension and lysed for 40 min, and the supernatant was collected by centrifugation at 12,000 rpm for 5 min at 4°C. The protein content (40 μg) of each sample was determined by using the BCA Protein Assay Kits (Thermo Scientific). Then, equal amounts of protein (12 μg/lane) were separated on 12% sodium dodecyl sulfate polyacrylamide gel electrophoresis (SDS-PAGE) and transferred to polyvinylidene fluoride (PVDF) membranes (Bio-Rad, Hercules, CA, United States). The membranes were blocked in 5% (w/v) skimmed milk powder in TBST (Tris buffered saline-0.1% Tween) for 2 h at 25°C, and then incubated with the following antibodies: rabbit polyclonal anti-β-actin antibody (1:1,000, Abcam, ab8227), rabbit monoclonal anti-PI3K antibody (1:1,000, Abcam, ab32089), rabbit polyclonal anti-AKT antibody (1:500, Abcam, ab8805), rabbit polyclonal anti-AKT (phospho T308) antibody (1:600, Abcam, ab38449), rabbit monoclonal anti-mTOR antibody (1:10,000, Abcam, ab134903), rabbit monoclonal anti-mTOR (phospho S2448) antibody (1:6,000, Abcam, ab109268), rabbit monoclonal anti-DNMT1 antibody (1:1,000, Abcam, ab188453). Before the ECL protocol (Amersham Biosciences, Piscataway, NJ, United States), the bands were visualized using horseradish peroxidase (HRP)-conjugated goat anti-rabbit IgG (1:5,000, Abcam, ab6721).

### Methylation-Specific Polymerase Chain Reaction

The methylation degree of Rnase6 gene promoter in peripheral blood from atherosclerotic patients or mice was determined by MSP. Conditions for polymerase chain reaction with Bisulfite-modified DNA: 95°C for 5 min, followed by 35 cycles of 95°C for 30 s, 64°C for 30 s, and 72°C for 30 s, with a final extension of 60°C for 30 min. Methylated (M) and unmethylated (U) PCR products were detected by gel electrophoresis. The relative intensity of bands was quantified with ImageJ (National Institutes of Health, Bethesda, MD, United States). The MSP primer sequences were shown below: Rnase6-MSP-M-F1 5′-TGG TTA AAT TTT AGA GGT CGG-3ʹ; Rnase6-MSP-M-R1 5′-CGA TTA TAA ACT CAT AAC TAA AAA CGA AA-3’; Rnase6-MSP-U-F1 5′-GGT TAA ATT TTA GAG GTT GG-3ʹ; Rnase6-MSP-U-R1 5′-TTA ACA ATT ATA AAC TCA TAA CT AAA AAC A-3ʹ.

### Statistical Analysis

All statistical analyses were performed by using the SPSS software (ver. 17.0; SPSS, Chicago, IL). The quantitative data derived from three independent experiments are expressed as mean ± standard error of mean (SEM). The Shapiro-Wilk test was used to verify that the data fit a normal distribution. Levene’s test was used to test for homogeneity of variance. A one-way analysis of variance (ANOVA) or Kruskal-Wallis non-parametric test was employed according to normal distribution and homogeneity of variance. The Kruskal Wallis test and post hoc analysis (Mann-Whitney *U* test) were used to assess pairwise differences between adjusted means. Values of *p* < 0.05 were considered statistically significant.

## Results

### Result 1 Upregulated Expression of Rnase6 in Peripheral Blood and Plaque Tissues of Atherosclerotic Patients

**FIGURE 1:**
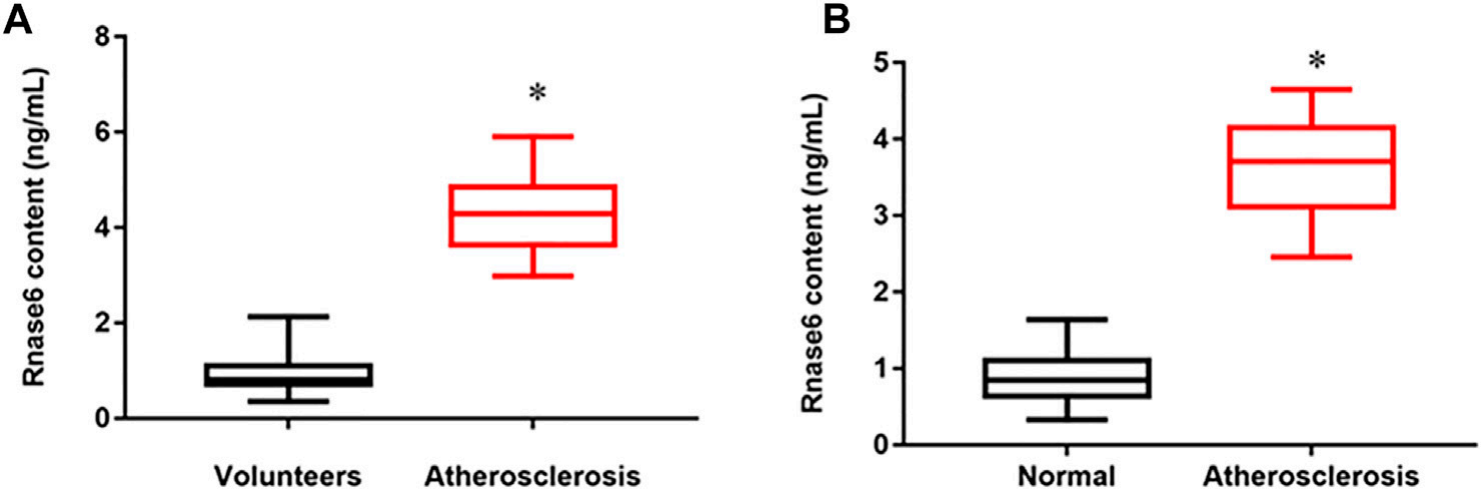
Upregulated expression of Rnase6 in peripheral blood and plaque tissue of atherosclerotic patients. **(A)**. Peripheral blood was collected from healthy volunteers and patients with atherosclerosis (N = 50, 25 volunteers and 25 patients). The secretion level of Rnase6 was detected by ELISA. **(B)**. The expression of Rnase6 in normal arterial wall and atherosclerotic tissue plaques were analyzed by ELISA. N = 6, *p < 0.01.

We collected peripheral blood from healthy volunteers and patients with atherosclerosis, and the results of ELISA showed that the expression of Rnase6 was significantly up-regulated in the peripheral blood of patients (Figure 1A). In addition, we obtained atherosclerotic plaque tissue and healthy arteries from patients undergoing carotid endarterectomy, and ELISA results suggested that Rnase6 expression in plaque tissue was also significantly increased (Figure 1B). The characteristics of subjects included in this study was shown in Table 1.

**TABLE 1:** Subjects’ characteristics.

Characteristic	Patients (n = 25)	Control (n = 25)
Sex		
Male	18	15
Female	7	10
Age		
Median (min-max)	54 (48–65)	56 (43–67)
<50	7	6
50–59	10	12
>60	8	7
BMI (kg/m^2^)	24 (22–26)	26 (23–29)
Smoking		
Current	19	18
Past	4	5
Never	2	2
Rutherford class		
III	9	–
IV	7	–
V	5	–
VI	4	–
Hypertension		
Yes	16	15
No	9	10
WBCs	6.89 ± 4.25	7.59 ± 2.36
Dyslipidemia	21	–

BMI, body mass index; WBC, white blood cells count.

### Result 2 Interference With Rnase6 Expression Inhibited OX-LDL-Induced Proliferation and Migration of MOVAS Cells

**FIGURE 2:**
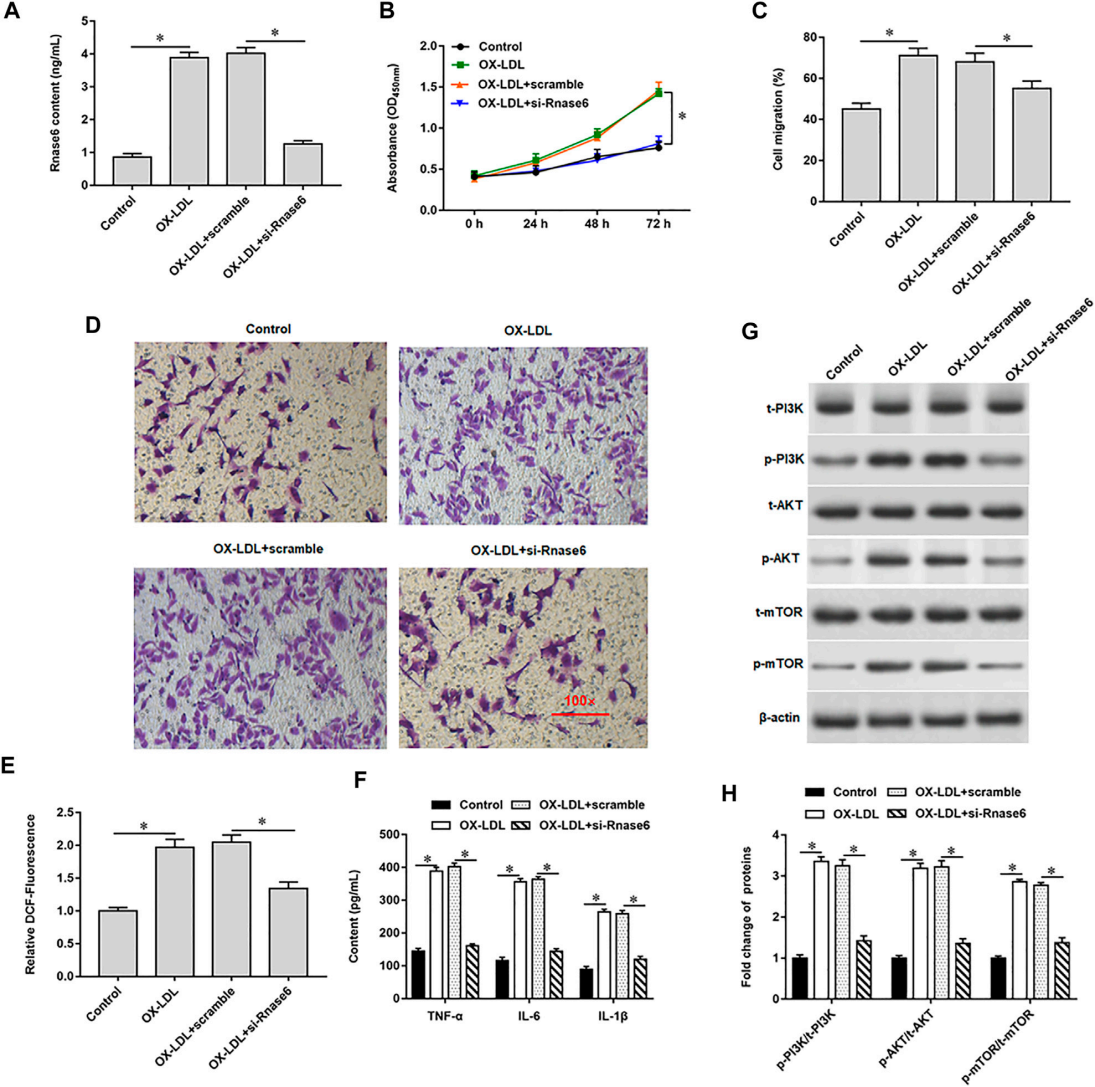
Interference with Rnase6 expression inhibited OX-LDL-induced proliferation and migration of MOVAS cells. MOVAS cells were induced with OX-LDL for 48 h, and scramble or si-Rnase6 (30 nM) were transfected in the cells, respectively. **(A)**. Relative expression of Rnase6 was detected by ELISA. **(B)**. CCK-8 assay was used to analyzed the proliferation of MOVAS cells. **(C,D)**. *Trans*-well assay was used to measure the cell migration. **(E).** ELISA was used to detect the content of ROS. **(F)**. Relative secretion levels of TNF-α, IL-6 and IL-1β were analyzed by ELISA kits. **(G,H)**. Fold change of signaling pathway-related proteins in cells were analyzed by Western blotting. β-actin was used as an internal reference. N = 6, *p < 0.01.

Our previous results showed that the expression of Rnase6 was up-regulated in the peripheral blood and plaque tissues of atherosclerotic patients. To study the regulatory role of Rnase6 in the development of AS more clearly, OX-LDL was used to induce mouse aortic vascular smooth muscle cells (MOVAS), and small interfering RNA of Rnase6 (si-Rnase6) was transfected into the cells. ELISA results showed that OX-LDL-induced upregulation of Rnase6 expression in MOVAS cells (Figure 2A), enhanced cell proliferation (Figure 2B) and migration (Figures 2C,D), increased reactive oxygen species (ROS) content (Figure 2E), and increased secretion of proinflammatory factors (Figure 2F), accompanied by increased phosphorylation levels of PI3K, AKT and mTOR proteins (Figures 2G,H). Conversely, interfering with Rnase6 expression significantly reversed the changes in cell behavior induced by OX-LDL.

### Result 3 The Presence of CpG Islands in the Promoter Region of Rnase6

**FIGURE 3:**
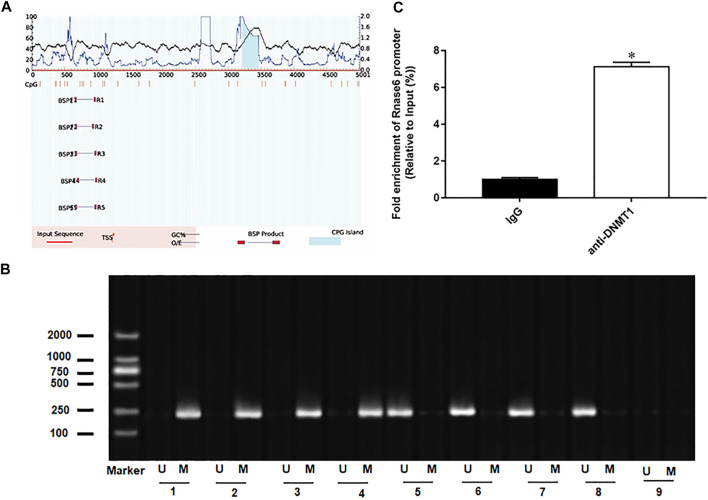
The presence of CpG islands in the promoter region of Rnase6. **(A)**. Cutting 5,000 bp upstream of the transcription start site of mouse Rnase6 gene as the promoter region, the distribution of CpG islands in the promoter region of Rnase6 gene was analyzed online using methylation prediction software (http://www.urogene.org/methprimer2/). **(B)**. MSP was used to detect the methylation status of Rnase6 in peripheral blood of healthy volunteers and patients with atherosclerosis. 1–4: healthy volunteers; 5–8: atherosclerosis patients; 9: blank control; U: unmethylated; m: methylated. **(C)**. Chromatin immunoprecipitation (ChIP) was used to verify the binding relationship between Rnase6 and DNA methyltransferase 1. N = 6, *p < 0.01.

In the progression of atherosclerosis, it may be accompanied by epigenetic changes of genes, leading to overexpression or significant inhibition of genes. Therefore, we continue to explore the mechanism of up-regulation of Rnase6 expression. We predicted CpG islands in the promoter region of the Rnase6 gene through an online biological database (http://www.urogene.org/methprimer2/) and found that there were indeed CpG islands upstream of its transcription start site (5,000 bp) (Figure 3A). Next, methylation-specific polymerase chain reaction (MSP) was used to detect the methylation status of Rnase6 in peripheral blood of healthy volunteers and patients with atherosclerosis. The results suggested that the Rnase6 gene was hypermethylated in the peripheral blood of healthy volunteers and hypomethylated in the peripheral blood of patients, which further confirmed that the up-regulation of Rnase6 expression was closely related to its decreased methylation level (Figure 3B). As the core enzyme catalyzing DNA methylation, DNA methyltransferase 1 (DNMT1) plays an important role in the methylation of CpG islands. ChIP results showed that was DNMT1 enriched in the promoter region of Rnse6 (Figure 3C).

### Result 4 Promoting Rnase6 Promoter Methylation Inhibited OX-LDL-Induced Proliferation and Migration of MOVAS Cells

**FIGURE 4:**
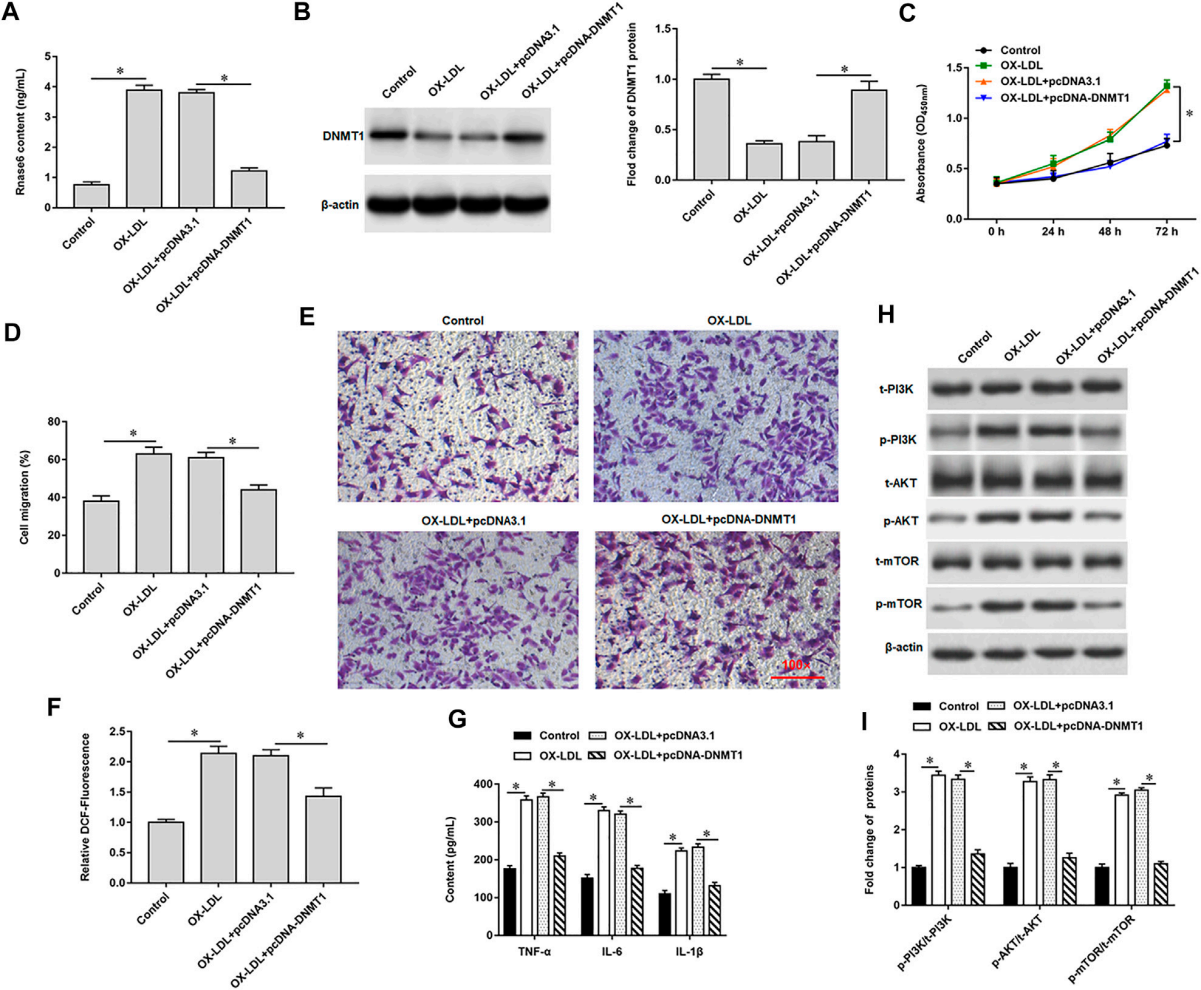
Promoting Rnase6 promoter methylation inhibited OX-LDL-induced proliferation and migration of MOVAS cells. MOVAS cells were induced with OX-LDL for 48 h, and pcDNA3.1 or pcDNA-DNMT1 (2.0 μg/ml) were transfected in the cells, respectively. **(A)**. ELISA was used to detected the expression of Rnase6. **(B)**. Western blotting was used to detect DNMT1 protein expression. **(C)**. Cell proliferation was analyzed by CCK-8 assay. **(D,E)**. *Trans*-well assay was used to measure the cell migration. **(F)**. ELISA was used to detect the content of ROS. **(G)**. Relative secretion levels of TNF-α, IL-6 and IL-1β were analyzed by ELISA kits. **(H,I)**. Fold change of signaling pathway-related proteins in cells were analyzed by Western blotting. β-actin was used as the loading control. N = 6, *p < 0.01.

We previously verified that DNMT1 could bind to Rnase6, suggesting that overexpression of DNMT1 in OX-LDL-induced MOVAS cells may affect Rnase6 promoter methylation and regulate its expression. As expected, transfection of pcDNA-DNMT1 significantly inhibited OX-LDL-induced Rnase6 expression in MOVAS cells (Figure 4A), decreased DNMT1 protein expression (Figure 4B), attenuated cell proliferation (Figure 4C) and migration (Figures 4D,E). Furthermore, ROS content (Figure 4F) and secretion level of proinflammatory factors (Figure 4G) in cells were also significantly reduced after overexpression of DNMT1. Western blotting results suggested that OX-LDL induction promoted the phosphorylation of PI3K signaling pathway-related proteins in cells, while transfection of pcDNA-DNMT1 reversed the expression level of phosphorylated proteins in cells (Figures 4H,I).

### Result 5 Hypomethylation of Rnase6 Promoter Activated PI3K/AKT/mTOR Signaling Pathway

**FIGURE 5:**
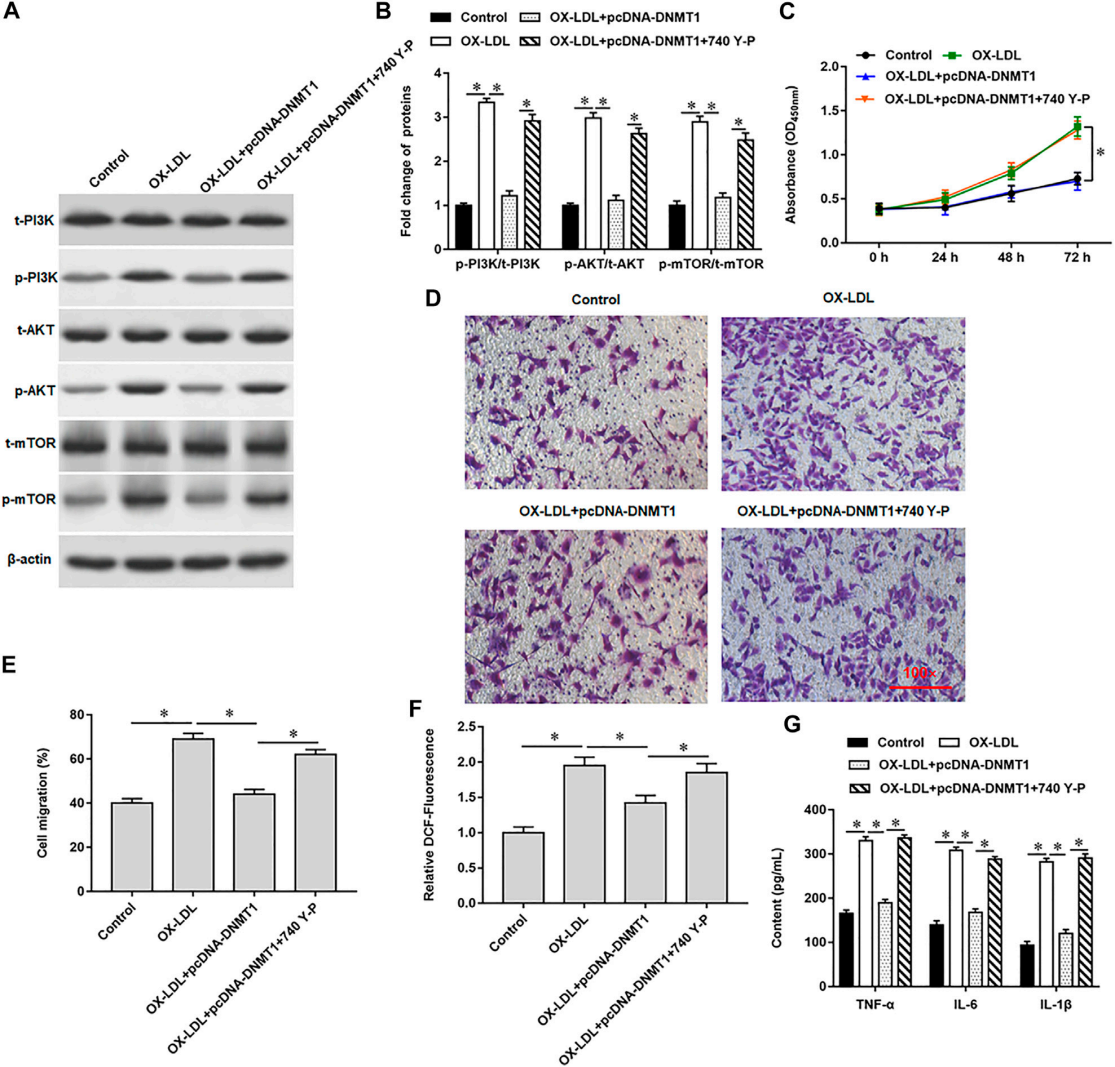
Hypomethylation of Rnase6 promoter activated PI3K/AKT/mTOR signaling pathway. MOVAS cells were induced with OX-LDL for 48 h, pcDNA-DNMT1 (2 μg/ml) was transfected into the cells, and the cells were intervened with PI3K agonist. **(A,B)**. Fold change of signaling pathway-related proteins in cells were analyzed by Western blotting. **(C)**. CCK-8 assay was used to analyzed the proliferation of MOVAS cells. **(D,E)**. *Trans*-well assay was used to measure the cell migration. **(F)**. ELISA was used to detect the content of ROS. **(G)**. Relative secretion levels of TNF-α, IL-6 and IL-1β were analyzed by ELISA kits. β-actin was used as an internal reference. N = 6, *p < 0.01.

Our previous results showed that overexpression of DNMT1 significantly reduced the expression of phosphorylated proteins in OX-LDL-induced MOVAS cells. Here, we introduced 740 Y-P, a PI3K agonist, to further validated the regulation of PI3K signal transduction system by hypomethylation of Rnase6 promoter. Western blotting results showed that 740 Y-P reversed the inhibitory effect of overexpressing DNMT1 on PI3K signaling pathway-related protein expression (Figures 5A,B). Moreover, the intervention of 740 Y-P re-enhanced the ability of cell proliferation (Figure 5C) and migration (Figure 5D,E), reduced ROS content (Figure 5F), and inhibited the secretion of proinflammatory factors (Figure 5G).

### Result 6 Hypomethylation of Rnase6 Promoter Aggravated Atherosclerosis in Mice

**FIGURE 6:**
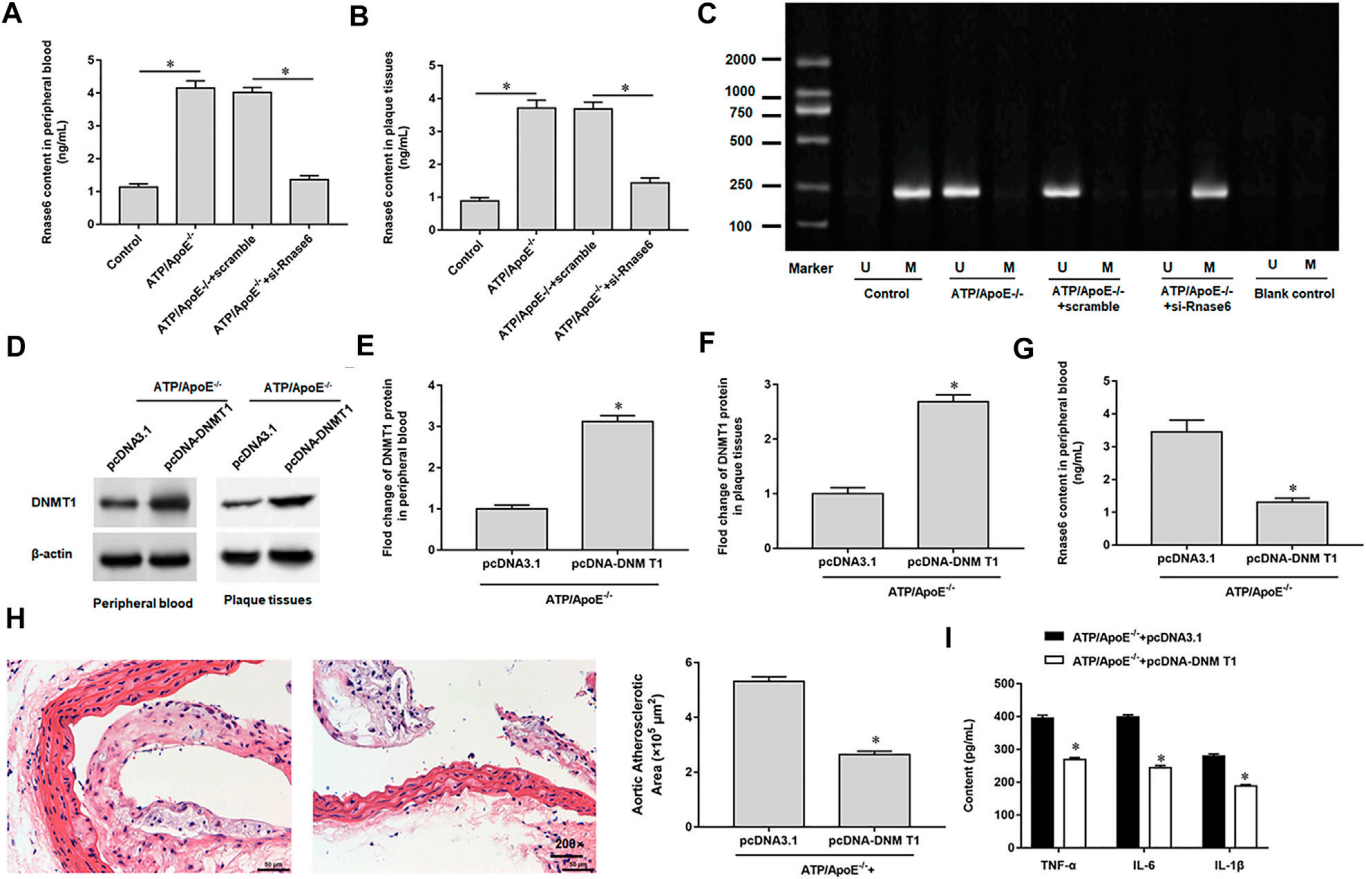
Hypomethylation of Rnase6 promoter aggravated atherosclerosis in mice. Sixteen ApoE^−/−^ mice were divided into control group (ApoE^−/−^ mice were given a basal diet), model group (ApoE^−/−^ mice were given a high-fat diet), scramble group (scramble was injected into the tail vein of high fat diet fed ApoE ^−/−^ mice, 0.5 mg/kg, twice a week) and si-Rnase6 group (si-Rnase6 was injected into the tail vein of high fat diet fed ApoE ^−/−^ mice, 0.5 mg/kg, twice a week), with four mice in each group. **(A,B)**. Peripheral blood and plaque tissues was collected from atherosclerosis mice, and the secretion level of Rnase6 was detected by ELISA. **(C)**. MSP was used to detect the methylation status of Rnase6 in peripheral blood of atherosclerosis mice. Eight ApoE^−/−^ mice were divided into pcDNA3.1 group (pcDNA3.1 was injected into the tail vein of high fat diet fed ApoE ^−/−^ mice, 200 μg/ml) and pcDNA-DNMT1 group (pcDNA-DNMT1 was injected into the tail vein of high fat diet fed ApoE^−/−^ mice, 200 μg/ml), with four mice in each group. **(D–F)**. The protein expression of DNMT1 in peripheral blood and plaque tissues was detected by Western blotting. **(G)**. The expression of Rnase6 in peripheral blood was detected ELISA. **(H)**. HE staining was performed on the plaque tissue of the aortic root of mice to observe the area of plaque formation. **(I)**. Relative secretion levels of TNF-α, IL-6 and IL-1β were analyzed by ELISA kits. N = 6, *p < 0.01.

We interfered with Rnase6 expression in apolipoprotein E (ApoE) knockout mice fed a high-fat diet and then collected peripheral blood and atherosclerotic plaques. ELISA results showed that the expression level of Rnase6 in peripheral blood and atherosclerotic plaques of atherosclerotic mice transfected with si-Rnase6 was significantly down-regulated compared with model mice (Figures 6A,B). In addition, MSP results showed that Rnase6 promoter was hypermethylated in peripheral blood of atherosclerotic mice whose Rnase6 expression was disturbed (Figure 6B).

We overexpressed DNMT1 in apolipoprotein E (ApoE) knockout mice fed a high-fat diet. Western botting results indicated increased DNMT1 expression in peripheral blood (Figures 6D,E) and sclerotic plaques (Figures 6D,F). ELISA analysis showed that Rnase6 protein expression levels were reduced in peripheral blood (Figure 6G). HE staining of mouse aortic root tissue revealed that overexpression of DNMT1 reduced the area of sclerotic plaque (Figure 6H). In addition, secretion of proinflammatory factors was reduced in the serum of atherosclerotic mice transfected with si-Rnase6 compared with model mice (Figure 6I).

## Discussion

Growing evidence suggests that epigenetic modifications play a crucial role in the regulation of gene expression, and DNA methylation occurring on CpG islands has been recognized as a universal mechanism for regulating gene expression in mammals (Heidari, et al., 2019). DNA promoter methylation has been widely reported as a novel epigenetic marker during atherosclerosis progression. Wei et al. reported that Smad7 expression was decreased in atherosclerotic plaques compared with normal arterial walls. Smad7 promoter DNA methylation levels at CpG unit 5.8.15.16 were increased in the peripheral blood of atherosclerosis patients compared with normal controls, and correlation analysis indicated that Smad7 promoter DNA methylation levels were positively correlated with homocysteine levels (Wei, L. et al., 2018). Hu et al. found that romidepsin attenuated TNF-α induced VCAM-1 expression and monocyte adhesion on the surface of HAECs by inhibiting HDAC1/2. Mechanistic studies suggested that down-regulation of VCAM-1 was attributed to decreased expression of the transcription factor GATA6. Romidepsin enhances STAT3 acetylation and its binding to DNMT1, leading to hypermethylation of promoter CpG rich regions at +140/+255. Blocking STAT3 acetylation at lys685 disrupts DNMT1-STAT3 interaction, decreases promoter methylation, and reverses the inhibitory effect of HDAC1/2 inhibition on GATA6 and VCAM-1 expression. These findings may provide a rationale for HDAC1/2-targeted therapy in atherosclerotic heart disease (Hu, et al., 2021). A survey of 30 patients with polycystic ovary syndrome reported that the methylation of TOX3 gene promoter in serum and granulosa cells of patients was significantly lower than that in healthy volunteers, suggesting that abnormal methylation of TOX3 gene has a significant promoting effect on the occurrence of polycystic ovary syndrome (Ning, et al., 2017). In addition, studies have shown that over expression of histone methyltransferase enhancer of Zeste homolog-2 lead to downregulation of ABCA1 gene in macrophage derived foam cells and accelerates atherosclerosis by promoting ABCA1 promoter methylation (Lv, et al., 2016). DNA methyltransferase inhibitor 5-aza-2′-deoxycytidine (5-aza-dC) was administered to LDL receptor knockout mice fed a high-fat diet, and it was found that 5-aza-dC-treated macrophages downregulated the expression of pro-inflammatory factors, attenuated the migration and adhesion of macrophages, thereby delaying the progression of atherosclerosis (Cao, et al., 2014).

Atherosclerosis is the main pathological basis of cardiovascular and cerebrovascular diseases, and its cardiovascular and cerebrovascular events are characterized by high mortality and disability rates. Therefore, starting from the cellular and molecular biological mechanisms of AS, exploring its therapeutic targets is of great significance for seeking drugs to effectively prevent and treat atherosclerosis. Rnase6, a protein belonging to the superfamily, is considered the only vertebrate specific enzyme known to have a wide range of physiological functions, including digestion, cytotoxicity, angiogenesis, male reproduction, and host defense. In this study, we found that the expression of Rnase6 was up-regulated in the peripheral blood and plaque tissues of atherosclerotic patients, but its promoter methylation was weakened, suggesting that abnormal Rnase6 promoter methylation is involved in the occurrence and development of atherosclerosis. Therefore, we promoted Rnase6 promoter methylation in OX-LDL-induced MOVAS cells, and the results suggested that enhancing Rnase6 promoter methylation inhibited the expression of Rnase6, attenuated the proliferation and migration ability of MOVAS cells, and reduced the secretion of proinflammatory factors and ROS content. Similarly, interfering with Rnase6 expression in ApoE knockout mice fed a high-fat diet, we found that Rnase6 gene methylation was enhanced in peripheral blood. In addition, promoting Rnase6 promoter methylation significantly inhibited the formation of plaque tissue and alleviated atherosclerosis in mice.

The phosphoinositide 3 kinase (PI3K) protein family is a dimer consisting of the regulatory subunit p85 and the catalytic subunit p110. When it binds to the growth factor receptor, it can change the protein structure of Akt and activate it, and activate or inhibit the activity of a series of downstream substrates by phosphorylation, thereby regulating the phenotypes of cell proliferation, differentiation, apoptosis and migration. Studies have reported that downregulation of Lnc00113 expression significantly inhibits the proliferation, survival and migration of vascular smooth muscle cells and endothelial cells, and the expression of phosphorylated proteins of PI3K, Akt and mTOR in cells is also inhibited (Yao, et al., 2018). Liu et al. demonstrated through *in vitro* and *in vivo* studies that quercetin can attenuate high fructose feeding-induced atherosclerosis in mice by inhibiting inflammation and cell apoptosis through ROS-regulated PI3K/AKT signaling pathway (Lu, et al., 2017). A study showed that the expression of miR-126 was decreased in OX-LDL-treated HUVECs, and overexpression of miR-126 reversed OX-LDL-induced HUVECs injury and impaired autophagic flux by blocking the PI3K/Akt/mTOR signaling pathway, thereby reducing the damage of atherosclerotic endothelial cells (Tang and yang, 2018).

In conclusion, our study confirmed that Rnase6 promoter hypomethylation accelerated the proliferation, migration and inflammatory infiltration of injured vascular smooth muscle cells and aggravated atherosclerosis in mice by activating the PI3K/AKT/mTOR signaling pathway, which provided more ideas for finding potential therapeutic targets for atherosclerosis.

## Data Availability

The original contributions presented in the study are included in the article/supplementary material, further inquiries can be directed to the corresponding author.

## Abbreviations

AS, atherosclerosis; ECs, endothelial cells; VSMCs, vascular smooth muscle cells; DNMT1, DNA methyltransferase 1; Rnase6, Ribonuclease 6; RNaseA, Ribonuclease A; MOVAS, mouse aortic vascular smooth muscle cells; OX-LDL, low-density lipoprotein; MSP, methylation-specific polymerase chain reaction; ApoE, apolipoprotein E; PI3K, phosphoinositide 3 kinase; SDS-PAGE, sodium dodecyl sulfate polyacrylamide gel electrophoresis; PVDF, polyvinylidene difluoride; HRP, horseradish peroxidase.

## References

[B1] Abdel-Hafiz H. A. Horwitz K. B. (2015). Role of Epigenetic Modifications in Luminal Breast Cancer. Epigenomics 7 (5), 847–862. 10.2217/epi.15.10 25689414 PMC4539290

[B2] Baumer Y. McCurdy S. Alcala M. Mehta N. Lee B.-H. Ginsberg M. H. (2017). CD98 Regulates Vascular Smooth Muscle Cell Proliferation in Atherosclerosis. Atherosclerosis 256, 105–114. 10.1016/j.atherosclerosis.2016.11.017 28012647 PMC5276722

[B3] Cao Q. Wang X. Jia L. Mondal A. K. Diallo A. Hawkins G. A. (2014). Inhibiting DNA Methylation by 5-Aza-2′-Deoxycytidine Ameliorates Atherosclerosis through Suppressing Macrophage Inflammation. Endocrinology 155 (12), 4925–4938. 10.1210/en.2014-1595 25251587 PMC4239421

[B4] Deng Y. Lei T. Li H. Mo X. Wang Z. Ou H. (2018). ERK5/KLF2 Activation Is Involved in the Reducing Effects of Puerarin on Monocyte Adhesion to Endothelial Cells and Atherosclerotic Lesion in Apolipoprotein E-Deficient Mice. Biochim. Biophys. Acta (Bba) - Mol. Basis Dis. 1864 (8), 2590–2599. 10.1016/j.bbadis.2018.04.021 29723698

[B5] Gao Z. F. Ji X. L. Gu J. Wang X. Y. Ding L. Zhang H. (2019). microRNA‐ 107 Protects against Inflammation and Endoplasmic Reticulum Stress of Vascular Endothelial Cells via KRT1‐dependent Notch Signaling Pathway in a Mouse Model of Coronary Atherosclerosis. J. Cel Physiol 234 (7), 12029–12041. 10.1002/jcp.27864 30548623

[B6] Guarrera S. Viberti C. Cugliari G. Allione A. Casalone E. Betti M. (2019). Peripheral Blood DNA Methylation as Potential Biomarker of Malignant Pleural Mesothelioma in Asbestos-Exposed Subjects. J. Thorac. Oncol. 14 (3), 527–539. 10.1016/j.jtho.2018.10.163 30408567

[B7] Heidari L. Ghaderian S. M. H. Vakili H. Salmani T. A. (2019). Promoter Methylation and Functional Variants in Arachidonate 5‐lipoxygenase and Forkhead Box Protein O1 Genes Associated with Coronary Artery Disease. J. Cel Biochem 120 (8), 12360–12368. 10.1002/jcb.28501 30825235

[B8] Hu C. Peng K. Wu Q. Wang Y. Fan X. Zhang D.-M. (2021). HDAC1 and 2 Regulate Endothelial VCAM-1 Expression and Atherogenesis by Suppressing Methylation of the GATA6 Promoter. Theranostics 11 (11), 5605–5619. 10.7150/thno.55878 33859766 PMC8039941

[B9] Jamebozorgi I. Majidizadeh T. Pouryaghoub G. Mahjoubi F. (2018). Aberrant DNA Methylation of Two Tumor Suppressor Genes, p14ARF and p15INK4b , after Chronic Occupational Exposure to Low Level of Benzene. Int. J. Occup. Environ. Med. 9 (3), 145–151. 10.15171/ijoem.2018.1317 29995020 PMC6466977

[B10] Lacey M. Baribault C. Ehrlich K. C. Ehrlich M. (2019). Atherosclerosis-associated Differentially Methylated Regions Can Reflect the Disease Phenotype and Are Often at Enhancers. Atherosclerosis 280, 183–191. 10.1016/j.atherosclerosis.2018.11.031 30529831 PMC6348116

[B11] Lang D. Lim B. K. Gao Y. Wang X. (2019). Adaptive Evolutionary Expansion of the Ribonuclease 6 in Rodentia. Integr. Zoolog. 14 (3), 306–317. 10.1111/1749-4877.12382 30688011

[B12] Li M. Liu Q. Lei J. Wang X. Chen X. Ding Y. (2017). MiR-362-3p Inhibits the Proliferation and Migration of Vascular Smooth Muscle Cells in Atherosclerosis by Targeting ADAMTS1. Biochem. Biophysical Res. Commun. 493 (1), 270–276. 10.1016/j.bbrc.2017.09.031 28890348

[B13] Lu X.-L. Zhao C.-H. Yao X.-L. Zhang H. (2017). Quercetin Attenuates High Fructose Feeding-Induced Atherosclerosis by Suppressing Inflammation and Apoptosis via ROS-Regulated PI3K/AKT Signaling Pathway. Biomed. Pharmacother. 85, 658–671. 10.1016/j.biopha.2016.11.077 27919735

[B14] Lv Y.-C. Tang Y.-Y. Zhang P. Wan W. Yao F. He P.-P. (2016). Histone Methyltransferase Enhancer of Zeste Homolog 2-Mediated ABCA1 Promoter DNA Methylation Contributes to the Progression of Atherosclerosis. PLoS One 11 (6), e0157265. 10.1371/journal.pone.0157265 27295295 PMC4905646

[B15] Nelli R. K. De Koster J. Roberts J. N. de Souza J. Lock A. L. Raphael W. (2019). Impact of Uterine Macrophage Phenotype on Placental Retention in Dairy Cows. Theriogenology 127, 145–152. 10.1016/j.theriogenology.2019.01.011 30695743

[B16] Ning Z. Jiayi L. Jian R. Wanli X. (2017). Relationship between Abnormal TOX3 Gene Methylation and Polycystic Ovarian Syndrome. Eur. Rev. Med. Pharmacol. Sci. 21 (9), 2034–2038. 28537684

[B17] Sun P. Li L. Liu Y. Z. Li G. Z. Xu Q. H. Wang M. (2019). MiR-181b Regulates Atherosclerotic Inflammation and Vascular Endothelial Function through Notch1 Signaling Pathway. Eur. Rev. Med. Pharmacol. Sci. 23 (7), 3051–3057. 10.26355/eurrev_201904_17587 31002155

[B18] Tang F. Yang T.-L. (2018). MicroRNA-126 Alleviates Endothelial Cells Injury in Atherosclerosis by Restoring Autophagic Flux via Inhibiting of PI3K/Akt/mTOR Pathway. Biochem. Biophysical Res. Commun. 495 (1), 1482–1489. 10.1016/j.bbrc.2017.12.001 29203244

[B19] Viafara-Garcia S. M. Gualtero D. F. Avila-Ceballos D. Lafaurie G. I. (2018). Eikenella Corrodenslipopolysaccharide Stimulates the Pro-atherosclerotic Response in Human Coronary Artery Endothelial Cells and Monocyte Adhesion. Eur. J. Oral Sci. 126 (6), 476–484. 10.1111/eos.12580 30357941

[B20] Wei L. Zhao S. Wang G. Zhang S. Luo W. Qin Z. (2018). SMAD7 Methylation as a Novel Marker in Atherosclerosis. Biochem. Biophysical Res. Commun. 496 (2), 700–705. 10.1016/j.bbrc.2018.01.121 29366786

[B21] Wu R. Wang L. Yin R. Hudlikar R. Li S. Kuo H. C. D. (2020). Epigenetics/epigenomics and Prevention by Curcumin of Early Stages of Inflammatory‐driven colon Cancer. Mol. Carcinog 59 (2), 227–236. 10.1002/mc.23146 31820492 PMC6946865

[B22] Xia Z. Gu M. Jia X. Wang X. Wu C. Guo J. (2018). Integrated DNA Methylation and Gene Expression Analysis Identifies SLAMF7 as a Key Regulator of Atherosclerosis. Aging 10 (6), 1324–1337. 10.18632/aging.101470 29905534 PMC6046250

[B23] Yao X. Yan C. Zhang L. Li Y. Wan Q. (2018). LncRNA ENST00113 Promotes Proliferation, Survival, and Migration by Activating PI3K/Akt/mTOR Signaling Pathway in Atherosclerosis. Medicine (Baltimore) 97 (16), e0473. 10.1097/md.0000000000010473 29668625 PMC5916647

[B24] Yu M.-H. Lin M.-C. Huang C.-N. Chan K.-C. Wang C.-J. (2018). Acarbose Inhibits the Proliferation and Migration of Vascular Smooth Muscle Cells via Targeting Ras Signaling. Vasc. Pharmacol. 103-105, 8–15. 10.1016/j.vph.2018.02.001 29432898

[B25] Zhao B. Wang D. Liu Y. Zhang X. Wan Z. Wang J. (2020). Six-Gene Signature Associated with Immune Cells in the Progression of Atherosclerosis Discovered by Comprehensive Bioinformatics Analyses. Cardiovasc. Ther. 2020, 1–13. 10.1155/2020/1230513 PMC7416237 32821283

